# A Case of Dysgraphia after Cerebellar Infarction Where Functional NIRS Guided the Task Aimed at Activating the Hypoperfused Region

**DOI:** 10.1155/2021/6612541

**Published:** 2021-06-23

**Authors:** Mutsumi Fujii, Kazumi Tanigo, Hirokazu Yamamoto, Keijyu Kikugawa, Masayuki Shirakawa, Miki Ohgushi, Takaaki Chin

**Affiliations:** ^1^Department of Physical Medicine and Rehabilitation, Hyogo Rehabilitation Center, Kobe, Japan; ^2^Department of Rehabilitation, Hyogo Rehabilitation Center, Kobe, Japan; ^3^Hyogo Rehabilitation Center, Kobe, Japan

## Abstract

**Background:**

Linguistic impairment following cerebellar lesions is characterized by a marked cerebellocerebral diaschisis with decreased perfusion in the left cerebral hemisphere.

**Case:**

We report on a 60-year-old right-handed French chef who presented with linguistic deficits following a right cerebellar infarction. Neurolinguistic examinations in the acute phase showed impaired graphomotor planning, especially for kanji (Japanese morphograms). Despite the absence of any structural damage to the supratentorial brain regions, a quantitative ^123^I-IMP SPECT study revealed a relative hypoperfusion, mainly around the left posterior middle temporal gyrus, considered to be a crossed cerebellar-cerebral diaschisis. We performed functional near-infrared spectroscopy (fNIRS) and observed that a picture card task could increase blood perfusion in the affected area. This task was as follows: once he saw a picture card depicting a dish, the patient had to list the ingredients that make up the dish. For example, he had to name vegetables, meat, and spices upon seeing a “curry” picture card. We added this task to his daily speech-hearing therapy regimen. In the chronic phase, we confirmed symptom amelioration in linguistic performance-paralleled reduction in the level of hypoperfusion on SPECT study. *Discussion*. This case is the first report of an fNIRS approach used to evaluate evidence-based prospective speech-hearing tasks by observing blood flow to the hypoperfused area of the cerebral cortex surface.

## 1. Introduction

Schmahmann and Sherman introduced the concept of Cerebellar Cognitive Affective Syndrome, defining a group of deficits characterized by impairment of executive functions, difficulties with spatial cognition, personality change, and language deficits such as agrammatism and dysprosody following cerebellar damage [1]. Moreover, the recent results of neuroanatomical, clinical, and neuroimaging studies have demonstrated that the cerebellum appears to be involved in the modulation of a broad spectrum of linguistic functions such as verbal fluency, word retrieval, syntax, reading, and writing [2].

In recent years, data supporting participation of the cerebellum in language have come from a number of functional neuroimaging studies such as positron emission tomography (PET), functional magnetic resonance imaging (fMRI), and single photon emission computed tomography (SPECT). These techniques are important because they represent the only relatively noninvasive means of monitoring neuronal activity in humans by directly measuring associated changes in blood flow and oxygenation [2]. SPECT studies demonstrated a decrease in blood flow in the left cerebral hemisphere after hemorrhage or infarction mostly in the right cerebellum [3–7]. The effects of diaschisis are well known, that is, hypometabolism in otherwise unaffected brain regions functionally connected to brain areas involved by the stroke [8]. These changes in hypoperfusion provide evidence for cortical involvement in dysgraphia after cerebellum stroke. However, it is unclear whether this hypoperfused area can be activated by some other sort of stimulation instead of word generation task.

Functional near-infrared spectroscopy (fNIRS) is another useful system to observe neuronal activation especially in a clinical setting. The advantages of fNIRS are as follows: it is relatively robust with regard to subject motion, it requires relatively little time for attachment with less onerous constraints, and it can be used in a sitting position [9]. Here we report a patient who presented with writing disruption after a right cerebellar infarction considered to be a result of crossed cerebellar-cerebral diaschisis on SPECT. Then we performed fNIRS study with the patient to determine a task that can stimulate the left cerebrum hypoperfused area shown by the SPECT study, and the task was added to the daily speech and language therapy (ST). This is the first case report where ST was associated with the fNIRS approach.

## 2. Case

### 2.1. History

**Figure 1:**
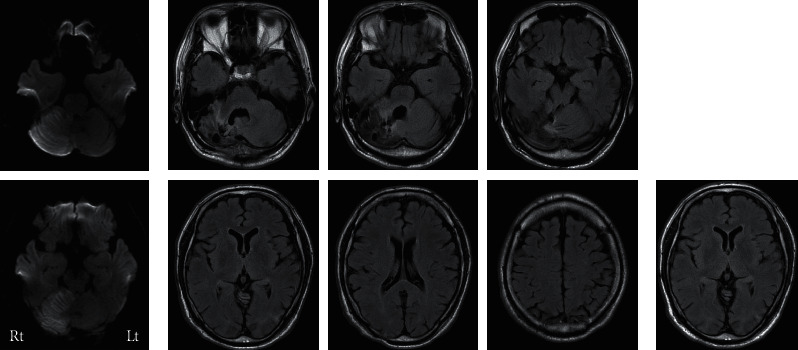
(a) Magnetic resonance (MR) images on day 0. Axial diffusion weighted images demonstrating a very new infarction in the right cerebellar hemisphere. Rt: right, Lt: left. (b) MR images on day 148. Axial FLAIR (fluid attenuated inversion recovery) MR images demonstrating no evidence of supratentorial damage without current infarction in the right cerebellar hemisphere. (c) A MR image 3 years after stroke onset. An axial FLAIR MR image demonstrating still no structural damage on the left posterior middle temporal gyrus.

**Figure 2:**
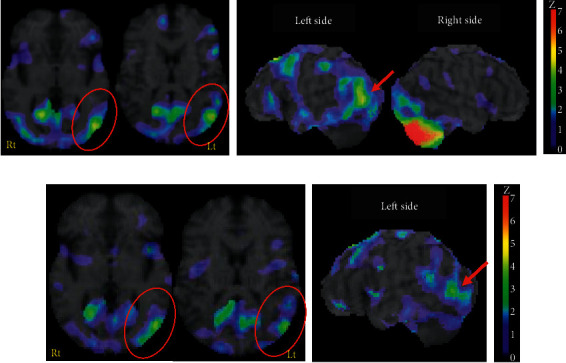
(a) A single photon emission computed tomography using ^123^I-N-isopropyl-p-iodoamphetamine (^123^I-IMP SPECT) perfusion scans on day 98. Compared with a standard normal database in perfusion findings, the *Z*-scores (SD) were calculated for each region and a regional *Z*-score of >2.0 was considered significant and indicated in green to red (compatible with the side color bar). The decreased perfusion was mainly observed in the left posterior middle frontal gyrus (pointed by red circles and arrows). (b) A following SPECT 3 years after stroke onset. The previous hypoperfusion showed an attenuation in both the area and degree on the left side view of this study.

A 60-year-old native Japanese-speaking right-handed man, with 12 years of education, was admitted to an emergency hospital with vertigo and postural instability. He was a French chef. Apart from atrial fibrillation without medication, his medical history was unremarkable. Neurologic examination on admission showed a diplopia, dysarthria, and right cerebellar ataxia. Magnetic resonance images (MRI) on brain (GE Healthcare Japan; Sigma HDxt 1.5T) revealed a new infarction in the right cerebellar hemisphere involving cortex and the medullary core due to the obstructions of the right superior cerebellar artery and the right posterior inferior cerebellar artery (Figure 1(a)). He underwent subtentorial open decompressive craniectomy for brain edema. He was transferred to our rehabilitation hospital on day 35. Neurological assessment revealed gait ataxia and right-sided hemiataxia. The score in the simple test for evaluating hand function (STEF) that was designed to evaluate the speed of manipulating (catching, pinching, and carrying) objects (10 different shapes and sizes) using an upper limb [10] was 62 out of a maximum 100 points for the right hand. Speech was slightly dysarthric. On neurological examination, writing kanji (Japanese morphograms, originally adopted from Chinese characters) words and naming were defective, but the dysgraphia was too pronounced to be explained by hemiataxia. Although no evidence of supratentorial damage was found on MRI (General Electric Company; Sigma Excite 1.5T) (Figure 1(b)), SPECT using ^123^I-N-isopropyl-p-iodoamphetamine (^123^I-IMP SPECT) perfusion scans (Toshiba Medical Systems Corporation; Symbia E) on day 98 revealed lower levels of perfusion, mainly in the left posterior middle temporal gyrus, left superior and middle temporal gyrus, and right cerebellar hemisphere (the current infarct area), than those observed in normal individuals (normal database findings) (Figure 2(a)). We proposed that hypoperfusion due to crossed cerebellar-cerebral diaschisis is a cause of his poor kanji writing.

### 2.2. Neurolinguistic and Neurocognitive Assessments

**Figure 3:**
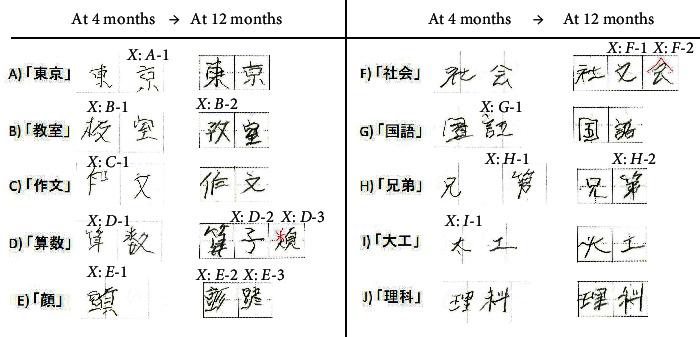
Samples of the patient's handwriting in the dictation ability between 4th and 12th month after onset. Kanji characters were chosen from the screening writing under the 4th grade test, (A) 「東京」 ([Tokyo], Tokyo), (B) 「教室」 ([kyoshitsu], classroom), (C)「作文」 ([sakubun], essay), (D) 「算数」 ([sansu], mathematics), (E) 「顔」 ([kao], face), (F)「社会」 ([shakai], society), (G)「国語」 ([kokugo], national language), (H)「兄弟」 ([kyodai], brother), (I)「大工」 ([daiku], carpenter), and (J)「理科」 ([rika], science). Underlined letters are kanji. Japanese pronunciation and meaning in English are noted. *X* is incorrect kanji letters. Red strokes are additional handwriting by a speech therapist. Phonologically related errors: *D*-2, *G*-1, *H*-1, and *H*-2; orthographically related errors: *A*-1, *B*-2, *C*-1, *D*-1, *D*-3, *E*-2, *E*-3, *F*-2, *F*-3, *G*-1, *H*-1, and *I*-1; semantically related errors: *B*-1 and *E*-1 (some were classified in combinations of these two types). Nonreal letters were as follows: *A*-1, *B*-2, *C*-1, *D*-1, *D*-3, *E*-2, *E*-3, *F*-3, *G*-1, and *H*-1.

ST was started on a daily basis, and the first in-depth neuropsychological investigations were performed 1.5 months after stroke. The language assessments of the Standard Language Test of Aphasia (SLTA), which is a formal neurolinguistic approach administered in Japanese [11], showed impaired writing, especially for kanji and verbal word fluency tests (Table 1). Articulation, repetition, oral reading, writing kana (the other type of syllabographic Japanese characters), and reading comprehension were almost normal. During a conversation, a listener was required to speculate and accordingly make a deduction in order to adequately comprehend the patient's response owing to the lack of subjects in his sentences or a frequent and abrupt change of topics. As verbal fluency tasks, we conducted category fluency tasks (semantic generation) and letter fluency tasks (phonological generation) in one minute. We regarded 15 or more word generation as normal. Results of verbal fluency tasks were moderately impaired: the number of words expressed aloud in semantic generation (“transportation” and “cooking tools”) were 7 and 2, and in phonological generation (started with “Sa” and “Ra”) were 4 and 6 at 4 months after the onset. Written kanji skills were also examined in more detail. In Japan, children between the ages of 6 (the 1^st^ grade) and 12 (the 6^th^ grade) are taught a total of 1006 kanji characters in elementary school, and the upper grade students gradually tend to learn more difficult kanji characters. The patient obtained a deficient score of 12/20 on a kanji screening writing test (that consisted of 1 or 2 kanji characters that were taught before the 5^th^ grade) at 4 months after the onset. His dysgraphia in the poor kanji writing constituted difficulty to recall correct letters with morphological, orthographical, or semantical errors, most of which resulted in nonwords (Figure 3). He was able to copy kanji characters in both spontaneous writing and dictation and the types of writing error were the same with both right and left hands. When it comes to the kanji character that he could not write correctly, it was difficult for him to recall it even after we offered hint of some radicals (the building components of kanji) or a part of the character. He also could not explain the shape of kanji characters or the stroke order, which means his letter recognition was impaired. Neurocognitive functions were evaluated by the trail making test and Kohs block design test at 1.5, 6, and 12 months after the onset and the Wechsler adult intelligence scale-III (WAIS-III) at 6 and 12 months after the onset (Table 2). Except for an attention disorder, the patient did not reveal prefrontal cortex dysfunction such as lack of spontaneity, loss of initiative, or flattened affect.

### 2.3. Rehabilitation Task Evaluation Using fNIRS

**Figure 4:**
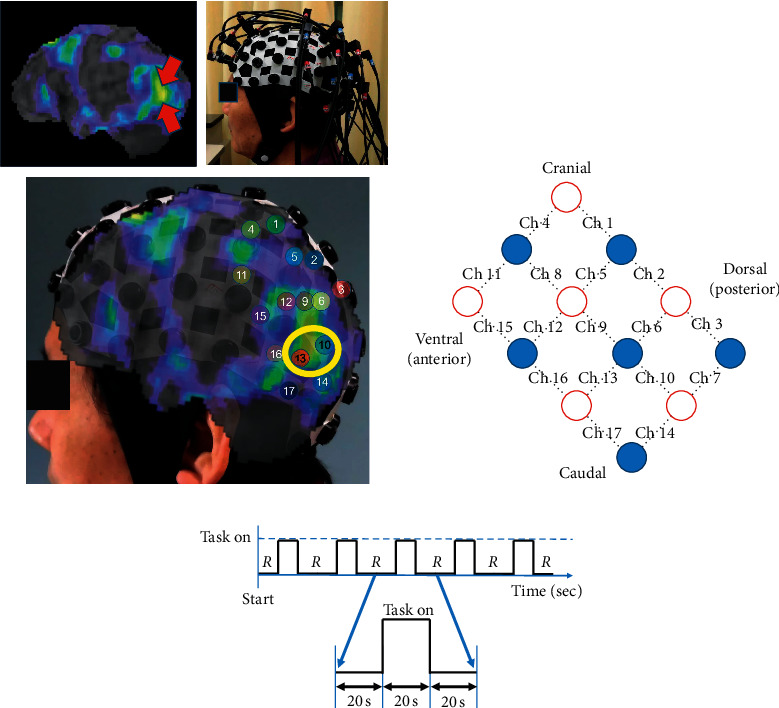
(a) Fusion map (lower photo) was comprised of left lateral side view of SPECT (upper left) and the patient's head photo in the fiber cap-type holder (upper right). The measurements were obtained from areas on both sides of the prefrontal and temporooccipital cortex (upper right). No. 10 and No. 13 channels (in a yellow circle) were considered to be receiving signals from the region of interest that corresponded to the hypoperfused area in the left posterior middle temporal gyrus. (b) Schematic figure of numbered channel arrangements that was indicated between the light detector (blue circle) and light source (red circle) on the left temporooccipital gyrus on fNIRS. (c) The procedure of tasks. The patient was instructed to empty his mind without movement for 20 sec, and then the operator prompted the patient to start a task. After a 20 sec task period, the patient was ordered to relax for another 20 sec: 5 cycles of 20 sec of rest, 20 sec of task, and 20 sec of rest were performed for averaging. For example, for the 5 times' task, we showed the patient the picture of curry, miso soup, ramen, a combination meal of rice and hamburger steak, and sukiyaki, respectively. R: rest period.

Since writing kanji words and naming did not improve 4 months after the onset, we performed fNIRS study to identify a task that would be able to recruit blood flow to the hypoperfused area in the left cerebral location. fNIRS is a noninvasive functional brain mapping method that uses near-infrared light. It can measure changes in regional cerebral blood flow induced by neural activity in the cerebral cortex based on absorbance at three wavelengths (780 nm, 805 nm, and 830 nm) according to the Beer-Lambert law. To detect task-related hemoglobin signal changes, a continuous-wave NIRS system (SMARTNIRS, Shimadzu Corp., Kyoto, Japan) with 17 light emitter fibers and 16 light detectors was employed, and data were analyzed by LABNIRS (Shimadzu Corp., Kyoto, Japan). We determined the locations of the emitter/detector probes and measurement channels. Every channel was expected to measure the changes at the midpoint between two probes and at a depth of 20–30 mm under the scalp. We concluded that No. 10 and No. 13 channels were recording signals from the regions of interest that corresponded to the hypoperfused area in the left posterior middle temporal gyrus; this conclusion was based on a fusion map generated using an image of the patient's head inside the fiber cap-type holder and a left-side SPECT view (Figure 4(a)). During the experiment, this holder was specifically placed for this patient so that it would position the probes at the same location every time. Channels corresponding to the left temporooccipital cortex are shown in Figure 4(b). The patient sat on a chair during the experiment. We considered the cortical oxygenated hemoglobin (oxy-Hb) signal increase as a real-time stimulatory response to a task [9]. After a long rest and the stabilization of the oxy-Hb level, we began a test. The operator ordered the patient to start the first task after 20 sec of resting. Before and after the 20 sec tasks, the patient was instructed to clear his mind and not move for 20 sec, and each 20 sec rest-task-rest cycle was repeated 5 times for averaging (Figure 4(c)).

**Figure 5:**
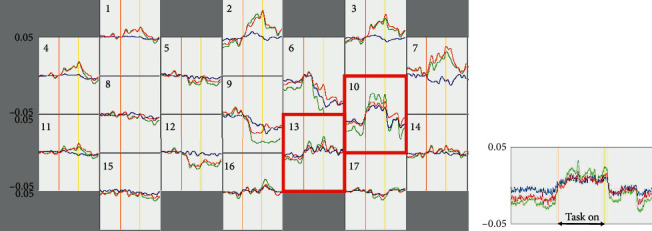
(a) Raw data after merging 5 continuous trials of the picture card task on fNIRS. Relative change in hemoglobin (Hb) (a.u.) is shown on the vertical axis, and a task was ongoing during the time period between the orange and yellow lines in the horizontal axis. The red, green, and blue lines indicate changes in oxy-Hb, total-Hb, and deoxy-Hb, respectively. No. 10 and No. 13 channels (surrounded by a red frame) were allocated to the region of interest according to the fusion map in Figure 4(a). (b) Group-averaged fNIRS recordings at No. 10 and No. 13 channels in response to the picture card task stimulation showing a rapid activation and deactivation corresponding to the picture task.

After trying several tests such as word imaging tasks or difficult kanji recalling tasks over a few days, we finally identified a workable picture card task that could increase blood perfusion in the hypoperfused area of the left posterior middle temporal gyrus, where we presumed to be the anatomical location of pure agraphia of kanji (Figure 5) [12]. The task was as follows: upon being shown a card that pictured a dish prepared with various ingredients, the patient had to think and name the ingredients of the dish, while remaining motionless. For example, he had to name most vegetables, meat, and spices that he expected to be contained in the dish after seeing a “curry” picture card. We conducted a comparison kanji writing test between one week before and just after the first picture card naming task. These two tests were totally different but the difficulty was intentionally the same. They consisted of 1 or 2 kanji characters which were taught in 3rd and 4th grade, and each pair of the kanji characters had the same number of strokes and the same importance level based on the Japanese pedagogical wordlists. The kanji writing score improved from 5 (one week before) to 8 out of 20 points after the first card naming task. Since the task took about only 5 minutes to perform but seemed to be effective, it was incorporated into the daily ST a few times a week.

### 2.4. Outcome

The patient was discharged from our hospital on day 204, walking independently with a slight stagger. One year after the stroke, an overall improvement in linguistic and cognitive function was observed (Tables 1 and 2). Results on verbal fluency tasks were improved: the number of words expressed aloud in semantic generation (“transportation” and “cooking tools”) were 10 and 8, and in phonological generation (started with “Sa” and “Ra”) were 4 and 9. Spontaneous and dictational writing of kanji letters also resulted in a perfect recovery in the SLTA. And when we reassessed his writing kanji ability using the same kanji screening test that was previously used 4 months after the onset, the correct numbers of kanji characters improved to 14/20 (Figure 3). Since similar types of writing errors persisted, we concluded that his deficiencies in kanji writing slightly remained although he had no difficulty in dysphasia in his regular life. Follow-up MRI did not show any additional damage (Figure 1(c)) and previous hypoperfused areas were attenuated on SPECT study after 3 years (Figure 2(b)). Written informed consent was obtained from this individual participant included in the study.

## 3. Discussion

Here we report a case where fNIRS test was used to confirm brain blood flow changes in the hypoperfused area as a reflection of task stimulation. This area was defined by the ^123^I-IMP SPECT study and resulted from crossed cerebellar-cerebral diaschisis. In the clinical practice, the assessment for impairment analysis of aphasia and the selection of corresponding tasks are performed by a trained speech and language therapist. This is the first report that an evidence-based task was selected by observing blood flow using fNIRS and was incorporated into the daily ST regiment of the patient.

Nine cases of dysgraphia after cerebellar hemorrhage or infarction have been reported to date (Table 3) [3–7, 13, 14]. Given the perfusion deficits in the structurally unaffected left cerebral hemisphere in most cases (including ours), crossed cerebellocerebral diaschisis has been identified as a possible pathophysiological mechanism to explain cerebellar involvement in dysgraphia. The follow-up period in most cases was 1 year or less, and it appears to be difficult to obtain a complete recovery in dysgraphia. We previously reported a patient with right cerebellar hemorrhage in kanji writing errors after as long as 7 years [14]. Her kanji dysgraphia was markedly improved but did not fully recover, suggesting even if no structural damage in the cerebrum can be seen after cerebellum stroke, appropriate rehabilitation therapy (including speech therapy) is essential to address the relevant symptoms.

Japanese writing system is biscriptal: it involves syllabographic kana and morphographic kanji. Every kana character has a consistent character-sound correspondence, whereas kanji characters have various degrees of character-sound correspondence. While most individual kana characters have no meaning, every kanji character conveys some meaning, even if it is not a word on its own [15]. This provides a basis for dissociation between residual ability for kana and kanji writing after stroke in Japanese patients. According to the anatomically based dual-route hypothesis for writing in response to dictation, agraphia of kanji can happen by a disruption of phonological pathways, orthographic pathways, interaction between phonology and orthography, and interaction between parietal graphemic area and frontal hand area [16]. These pathways include the primary auditory cortex (Heschl's gyri), the posterior superior temporal gyrus (Wernicke's area), the supramarginal gyrus, the angular gyrus and the adjoining lateral occipital gyri, the posterior inferior temporal cortex (Brodmann Area 37), and the posterior middle and inferior frontal gyri (Areas 44/45 and 6). Although the patient showed slight kana writing mistakes of kana writing competence at 1.5 months in SLTA (Table 1), the symptom nearly disappeared in the next few weeks and then there remained certain pure kanji dysgraphia. Sakurai et al. reported that agraphia (without alexia) for kanji arose from an isolated lesion in the left posterior middle temporal gyrus, where the visual images of words or output from the image were considered to be processed [17]. We concluded that the culprit lesion for dysgraphia in our case should be coming from the dysfunction in the left posterior middle temporal gyrus. The region was also shown as a main hypoperfused area in the SPECT study.

Although fNIRS contains some strengths and limitations compared to other neuroimaging methods like functional magnetic resonance imaging in terms of temporal and spatial resolution, it is the most convenient and useful approach to supply blood perfusion information when the region of interest is limited to the cortical surface of the cerebrum [18]. Since the signals on the hypoperfused area in the posterior middle temporal gyrus was considered to correspond to channels No. 10 and No. 13 of fNIRS, we concentrated on their oxy-Hb signal trends during the task. First, we observed that more complicated kanji recall could increase oxy-Hb level in this region of interest (unpublished data). However, this task was too hard to administer because it loaded a substantial mental pressure for the dysgraphia patient to keep imaging increasingly difficult kanji letters. In fact, the patient did not want to deal with this task. In order to ensure that tasks can be administered on a daily basis, training tasks should avert excess effort for the patient. We had to set up alternative training task. We identified a good picture task wherein the patient looked at a card on which a type of dish consisting of various ingredients was depicted. Upon seeing the picture, the patient was requested to name food materials inside the dish. We considered the reasons behind this task being able to stimulate the left posterior middle temporal hypoperfused area. This region is thought to play a key role in both controlled aspects of semantic retrieval and the comprehension of events, relations, and actions [19]. It also supports controlled retrieval of weak conceptual combinations and associations [20]. The central role of the task is thought to be the work that we had the patient to perform when retrieving category-related objects from the picture (that is a categorically superordinate theme), after identifying the object illustrated on it. We also considered that the card task was not so hard for the patient, but sufficient to stimulate the left posterior middle temporal gyrus, in terms of the retrieval of relevant thematic associations. The patient accepted this food picture card task eagerly because he was a chef. And it is conceivable that this task could contribute to an amelioration in kanji dysgraphia as well as verbal fluency.

His writing and word fluency impairment were found to be attenuated one year after the onset. And we confirmed this improvement in linguistic performance paralleled reduction in the level of hypoperfusion. Since this attenuation had been improving even after 4 months of onset, we consider that the picture card task may make a favorable contribution to attenuate his impairment. To clarify this topic, we will have to accumulate kanji dysgraphia cases caused by the crossed cerebellar-cerebral diaschisis and evaluate rehabilitation effects in the future.

## 4. Conclusion

Here we report a case of linguistic deficits following a right cerebellar hemisphere infarction considered to be a result of crossed cerebellar-cerebral diaschisis. A quantified ^123^I-IMP SPECT study revealed hypoperfusion mainly around the left posterior middle temporal gyrus, which we considered was a plausible cause of his kanji dysgraphia. fNIRS allowed us to identify a picture card task that could increase blood perfusion in the hypoperfused area, so that the task could be incorporated in the daily ST regimen of the patient. This is the first report of a plausible evidence-based task for such a diaschisis condition, which was selected by observing blood flow using fNIRS, and which might contribute to attenuate dysgraphia.

## Figures and Tables

**Table 1:** The scores of the Japanese standard language test of aphasia (SLTA) at 1.5 months and 12 months after the onset.

Language modality	Test	Scores in 1.5 mo	Six-stage assessment on 1.5 mo						Scores in 1 yr	Six-stage assessment on 1 yr					
			6	5	4	3	2	1		6	5	4	3	2	1
Auditory comprehension	1, auditory word recognition	100	10	0	NA	0	NA	0	100	10	0	NA	0	NA	0
	2, sentence comprehension	100	10	0	NA	0	NA	0	100	8	2	NA	0	NA	0
	3, following verbal commands	90	9	0	1	0	0	0	90	9	0	1	0	0	0
	4, kana letter discrimination	100	10	0	NA	0	NA	0	100	10	0	NA	0	NA	0

Oral expression	5, object naming	90	17	1	0	0	0	2	90	18	0	0	1	0	1
	6, word repetition	100	10	0	0	0	0	0	100	10	0	0	0	0	0
	7, capability of explaining behaviors	100	8	2	0	0	0	0	100	8	2	0	0	0	0
	8, capability of explaining the content of comics	100	Stage 6						100	Stage 6					
	9, sentence repetition	80	4	0	1	0	0	0	100	5	0	0	0	0	0
	10, word fluency (listing)	73	11						87	13					
	11, reading aloud kanji words	100	5	0	0	0	0	0	100	5	0	0	0	0	0
	12, reading aloud kana letters	100	10	0	NA	NA	0	0	100	10	0	NA	NA	0	0
	13, reading aloud kana words	100	5	0	0	0	0	0	100	5	0	0	0	0	0
	14, reading aloud short sentences	100	5	0	0	0	0	0	100	4	1	0	0	0	0

Reading comprehension	15, matching written kanji word and picture	100	10	0	0	0	0	0	100	10	0	0	0	0	0
	16, matching written kana word and picture	100	9	1	0	0	0	0	100	10	0	0	0	0	0
	17, matching sentence and picture	100	7	3	NA	0	NA	0	90	9	0	NA	1	NA	0
	18, following written commands	80	6	2	1	0	0	1	90	9	0	0	1	0	0

Writing competence	19, writing kanji words	40	2	0	0	1	1	1	100	4	1	0	0	0	0
	20, writing kana words	80	1	3	0	1	0	0	100	3	2	0	0	0	0
	21, writing the story of a cartoon	60	Stage 4						80	Stage 5					
	22, dictating kana letters	100	10	0	0	0	0	0	100	10	0	0	0	0	0
	23, dictating kanji words	40	1	1	2	0	1	0	100	5	0	0	0	0	0
	24, dictating kana words	80	4	0	0	0	1	0	100	5	0	0	0	0	0
	25, dictating short sentences	100	1	4	0	0	0	0	100	5	0	0	0	0	0

Calculation	26, simple mathematical calculation	75	15						85	17					

The maximal score is 100 in every test. The score is considered 100% when the number of words reaches 15 words in “10, word fluency (listing).” Both stages 6 (perfect) and 5 (sluggish but good) in the six-stage assessment are considered “correct” in the score part, and stage 4 (a certain amount of error) to 1 (wrong answer) in the six-stage assessment are “incorrect” in the score part. The patient did not show any discontinuation over the tests. NA: not applicable.

**Table 2:** Neurocognitive functions were evaluated by the trail making test, Kohs block design test at 1.5, 6, and 12 months after the onset, and the Wechsler adult intelligence scale-III (WAIS-III) at 6 and 12 months after the onset.

Period from onset	1.5 months	6 months	12 months	Normal range
*Trail making test*				
Part A (sec)	125	88	91	157.6 ± 65.8
Part B (sec)	234	145	100	216.2 ± 84.7
Kohs block design test	108 (IQ115)	121 (IQ112)	128 (IQ119)	

*Wechsler adult intelligence scale-III*				
Total IQ (TIQ)		77	85	
Verbal IQ (VIQ)		78	82	
Performance IQ (PIQ)		80	91	
Verbal comprehension (VC)		88	92	
Perceptual organization (PO)		97	101	
Working memory (WM)		74	79	
Processing speed (PS)		66	66	

**Table 3:** Case reports of dysgraphia in patients with cerebellar stroke.

Author (year)	Age	Sex	Handedness	Lesion side	Disease	Putative mechanism	Follow-up	Outcome
Marien et al. [3]	73	Male	Right	Right	Infarction	Crossed cerebellocerebral diaschisis	1 year	Slightly amelioration of aphasic syndrome
Gasparini et al. [13]	51	Male	Right	Right	Infarction	The cerebellum's direct involvement (not showing any supratentorial abnormalities of perfusion distribution)	NA	NA
Marien et al. [4]	72	Male	Right	Right	Hemorrhage	Crossed cerebellocerebral diaschisis	6 months	Apraxic symptoms persisted in writing
Marien et al. [5]	58	Male	Right	Right	Infarction	Crossed cerebellocerebral diaschisis	4 weeks	Similar in writing to dictation
Fukunaga and Tokuda [6]	74	Male	Right	Right	Hemorrhage	Crossed cerebellocerebral diaschisis	3 months	Markedly improved
De Smet et al. [7]	74	Male	Right	Right	Hemorrhage	Crossed cerebellocerebral diaschisis	4 months	Amelioration
De Smet et al. [7]	86	Female	Right	Bilateral	Infarction	Same side hypoperfusion	6 months	Markedly improved
De Smet et al. [7]	76	Male	Right	Right	Infarction	Crossed cerebellocerebral diaschisis	NA (deceased)	NA
Fujii et al. [14]	48	Female	Right	Right	Hemorrhage	Crossed cerebellocerebral diaschisis	7 years	Markedly improved
Our case (2021)	60	Male	Right	Right	Infarction	Crossed cerebellocerebral diaschisis	1 year	Amelioration

NA: not applicable.

## Data Availability

No data were used to support the study.

## References

[1] Schmahmann J. D., Sherman J. C. (1998). The cerebellar cognitive affective syndrome. *Brain*.

[2] Murdoch B. E. (2010). The cerebellum and language: historical perspective and review. *Cortex*.

[3] Mariën P., Saerens J., Nanhoe R. (1996). Cerebellar induced aphasia: case report of cerebellar induced prefrontal aphasic language phenomena supported by SPECT findings. *Journal of the Neurological Sciences*.

[4] Mariën P., Verhoeven J., Brouns R., De Witte L., Dobbeleir A., De Deyn P. P. (2007). Apraxic agraphia following a right cerebellar hemorrhage. *Neurology*.

[5] Mariën P., Baillieux H., De Smet H. J. (2009). Cognitive, linguistic and affective disturbances following a right superior cerebellar artery infarction: a case study. *Cortex*.

[6] Fukunaga N., Tokuda Y. (2011). A case of cerebellar cognitive affective syndrome exhibiting paragraphia. *The Japanese Journal of Rehabilitation Medicine*.

[7] De Smet H. J., Engelborghs S., Paquier P. F., De Deyn P. P., Mariën P. (2011). Cerebellar-induced apraxic agraphia: a review and three new cases. *Brain and Cognition*.

[8] Shih W.-J., Huang W.-S., Milan P. P. (2006). F-18 FDG PET demonstrates crossed cerebellar diaschisis 20 years after stroke. *Clinical Nuclear Medicine*.

[9] Mihara M., Miyai I., Hattori N. (2012). Neurofeedback using real-time near-infrared spectroscopy enhances motor imagery related cortical activation. *PLoS One*.

[10] Shindo K., Oba H., Hara J., Ito M., Hotta F., Liu M. (2015). Psychometric properties of the simple test for evaluating hand function in patients with stroke. *Brain Injury*.

[11] Brain Function Test Committee of the Japanese Society for Higher Brain Dysfunction (2003). *Standard Language Test for Aphasia (SLTA)*.

[12] Soma Y., Sugishita M., Kitamura K., Maruyama S., Imanaga H. (1989). Lexical agraphia in the Japanese language. *Brain*.

[13] Gasparini M., Piero V. D., Ciccarelli O., Cacioppo M. M., Pantano P., Lenzi G. L. (1999). Linguistic impairment after right cerebellar stroke: a case report. *European Journal of Neurology*.

[14] Fujii M., Tanigo K., Hayakawa M. (2018). A Case of Dysgraphia with Right Cerebellum Infarction Seven Years after Onset.

[15] Sato H. (2015). Do different orthographies share the same mechanisms of reading? a review of research on and models for Japanese acquired dyslexia. *Aphasiology*.

[16] Sakurai Y., Onuma Y., Nakazawa G. (2007). Parietal dysgraphia: characterization of abnormal writing stroke sequences, character formation and character recall. *Behavioural Neurology*.

[17] Sakurai Y., Mimura I., Mannen T. (2008). Agraphia for kanji resulting from a left posterior middle temporal gyrus lesion. *Behavioural Neurology*.

[18] Scarapicchia V., Brown C., Mayo C., Gawryluk J. R. (2017). Functional magnetic resonance imaging and functional near-infrared spectroscopy: insights from combined recording studies. *Frontiers in Human Neuroscience*.

[19] Davey J., Thompson H. E., Hallam G. (2016). Exploring the role of the posterior middle temporal gyrus in semantic cognition: integration of anterior temporal lobe with executive processes. *Neuroimage*.

[20] Thompson H., Davey J., Hoffman P. (2017). Semantic control deficits impair understanding of thematic relationships more than object identity. *Neuropsychologia*.

